# Common Genetic Variation, Residential Proximity to Traffic Exposure, and Left Ventricular Mass: The Multi-Ethnic Study of Atherosclerosis

**DOI:** 10.1289/ehp.0901535

**Published:** 2010-03-22

**Authors:** Victor C. Van Hee, Sara D. Adar, Adam A. Szpiro, R. Graham Barr, Ana Diez Roux, David A. Bluemke, Lianne Sheppard, Edward A. Gill, Hossein Bahrami, Christina Wassel, Michele M. Sale, David S. Siscovick, Jerome I. Rotter, Stephen S. Rich, Joel D. Kaufman

**Affiliations:** 1 Department of Environmental and Occupational Health Sciences; 2 Department of Medicine; 3 Department of Epidemiology and; 4 Department of Biostatistics, University of Washington, Seattle, Washington, USA; 5 Department of Medicine and; 6 Department of Epidemiology, Columbia University Medical Center, New York, New York, USA; 7 Department of Epidemiology, University of Michigan, Ann Arbor, Michigan, USA; 8 Radiology and Imaging Sciences, National Institutes of Health, Department of Health and Human Services, Bethesda, Maryland, USA; 9 Department of Epidemiology, Johns Hopkins School of Public Health, Baltimore, Maryland, USA; 10 Department of Cardiology, Johns Hopkins School of Medicine, Baltimore, Maryland, USA; 11 Department of Epidemiology, University of Minnesota, Minneapolis, Minnesota, USA; 12 Department of Medical Genetics, Cedars-Sinai Medical Center, Los Angeles, California, USA; 13 Center for Public Health Genomics, University of Virginia, Charlottesville, Virginia, USA

**Keywords:** *AGTR1*, *ALOX15*, cardiac structure, cardiac MRI, gene-environment interactions, left ventricular mass, traffic, air pollution

## Abstract

**Background:**

Elevated left ventricular mass (LVM) is a strong predictor of negative cardiovascular outcomes, including heart failure, stroke, and sudden cardiac death. A relationship between close (< 50 m compared with > 150 m) residential proximity to major roadways and higher LVM has previously been described, but the mechanistic pathways that are involved in this relationship are not known. Understanding genetic factors that influence susceptibility to these effects may provide insight into relevant mechanistic pathways.

**Objective:**

We set out to determine whether genetic polymorphisms in genes affecting vascular and autonomic function, blood pressure, or inflammation influence the relationship between traffic proximity and LVM.

**Methods:**

This was a cross-sectional study of 1,376 genotyped participants in the Multi-Ethnic Study of Atherosclerosis, with cardiac magnetic resonance imaging performed between 2000 and 2002. The impact of tagged single-nucleotide polymorphisms (tagSNPs) and inferred haplotypes in 12 candidate genes (*ACE*, *ADRB2*, *AGT*, *AGTR1*, *ALOX15*, *EDN1*, *GRK4*, *PTGS1*, *PTGS2*, *TLR4*, *VEGFA*, and *VEGFB*) on the relationship between residential proximity to major roadways and LVM was analyzed using multiple linear regression, adjusting for multiple potential confounders.

**Results:**

After accounting for multiple testing and comparing homozygotes, tagSNPs in the type 1 angiotensin II receptor (*AGTR1*, rs6801836) and arachidonate 15-lipoxygenase (*ALOX15*, rs2664593) genes were each significantly (*q* < 0.2) associated with a 9–10% difference in the association between residential proximity to major roadways and LVM. Participants with suboptimal blood pressure control demonstrated stronger interactions between *AGTR1* and traffic proximity.

**Conclusions:**

Common polymorphisms in genes responsible for vascular function, inflammation, and oxidative stress appear to modify associations between proximity to major roadways and LVM. Further understanding of how genes modify effects of air pollution on CVD may help guide research efforts into specific mechanistic pathways.

Long-term exposure to ambient air pollution has been associated with cardiovascular morbidity and mortality in numerous epidemiologic studies (Dockery et al. 1993; Miller et al. 2007; Pope et al. 2004), but the mechanisms responsible for its observed effects remain poorly understood (Bhatnagar 2006). A variety of hypothesized pathways have been implicated in human and animal studies, including an effect of air pollution on endothelial function and vascular tone, autonomic function, thrombosis, blood pressure, and oxidative stress. Recent reviews of the studies implicating these pathways have been published (Brook 2007; Mills et al. 2009; Pope and Dockery 2006; Simkhovich et al. 2008).

Among studies that have demonstrated increased cardiovascular morbidity and mortality associated with air pollution exposures, traffic-related air pollution in particular has been increasingly recognized as an important source of cardiovascular disease (CVD)-modifying exposures. Recent work in the Multi-Ethnic Study of Atherosclerosis (MESA) has demonstrated a link between close (< 50 m) residential major roadway proximity, an indicator of high traffic exposure, and higher left ventricular mass (LVM) index (Van Hee et al. 2009). Increased LVM is associated with the subsequent development of depressed ejection fraction (Drazner et al. 2004), and left ventricular hypertrophy is associated with heart failure, arrhythmia, and sudden cardiac death (Bluemke et al. 2008; Levy et al. 1990). Efforts to elucidate the ways in which air pollution might relate to higher LVM may provide not only greater understanding of the underlying CVD process itself but also potential strategies for intervention.

Several genes and genetic pathways have been noted to have important functions in the postulated mechanisms responsible for the impact of air pollution on CVD. Components of the renin–angiotensin system, including angiotensinogen (*AGT*), angiotensin-converting enzyme (*ACE*), and type 1 angiotensin II receptor (*AGTR1*) genes, have well-described impacts on inflammation, blood pressure, and vasoconstriction (Gradman 2009) that are mediated in part via G protein–coupled receptor kinases (GRKs), including *GRK4* (Felder and Jose 2006). Prostaglandin-endoperoxide synthase 1 and 2 genes (*PTGS1* and *PTGS2*), as well as 15-lipoxygenase gene (*ALOX15*), play important roles in vascular inflammation and oxidative stress that leads to CVD (Dogné et al. 2005; Mochizuki and Kwon 2008; Wittwer and Hersberger 2007). Endothelin-1 (EDN1) is a potent vasoconstrictor that activates signaling pathways leading to atherosclerosis (Ivey et al. 2008). The beta-2 adrenergic receptor (ADRB2) affects cardiac autonomic function and the development of heart failure (Triposkiadis et al. 2009). Together with ALOX15, vascular endothelial growth factors A and B (VEGFA and VEGFB) promote vascular growth, which contributes to the development of atherosclerotic plaque and plaque instability (Mochizuki and Kwon 2008; Sluimer and Daemen 2009). Toll-like receptor 4 (TLR4) affects the inflammatory response that influences initiation and progression of atherosclerosis (Pasterkamp et al. 2004).

We conducted a study to determine whether polymorphisms in these prespecified genes involved in the regulation of vascular tone, blood pressure, autonomic function, and oxidative stress modify the previously observed relationship between proximity to major roadways and LVM in MESA.

## Materials and Methods

### Population and sampling

MESA is a prospective cohort study designed to examine the progression of subclinical CVD; it enrolled 6,814 men and women 44–85 years of age who were free of clinical CVD at entry. The participants were recruited from six U.S. communities: Baltimore, Maryland; Chicago, Illinois; Forsyth County, North Carolina; Los Angeles County, California; Northern Manhattan, New York; and St. Paul, Minnesota. Details of the sampling, recruitment, and data collection have been previously reported (Bild et al. 2002). A subcohort of 2,880 unrelated MESA subjects were selected for genetic studies from subjects who gave informed consent for DNA extraction and use in genetic studies and had sufficient DNA available for this study. Priority was given to subjects who were included in a 1,000-person subset of participants with additional blood biomarker measurements, supplemented by random selection from remaining participants to fulfill balanced ethnic group representation (720 African American, 720 Hispanic, 720 Chinese, and 720 European American) and equality by sex.

For the present study, we included all participants in the genetic study database (*n* = 2,847) who underwent cardiac magnetic resonance imaging (MRI) at examination 1 (*n* = 2,152), had accurate address information for exposure assignment, and represented the groups with the greatest contrast in traffic proximity exposure (living within 50 m compared with ≥ 150 m from a major roadway) and the largest LVM association from our prior study (Van Hee et al. 2009), leaving 1,376 participants for analysis.

### Study procedures

Medical history, address, anthropometric measurements, physical examination, and laboratory data for the present study were taken from the first examination of the MESA cohort, July 2000 to August 2002 (Bild et al. 2002). Information about age, sex, ethnicity, and medical history were obtained by questionnaires administered at the screening and the first examination. Institutional review boards at all participating centers approved the protocol, ensuring conformance with the Declaration of Helsinki, and all participants gave informed consent.

#### Geocoding and exposure assessment

Participants’ residential addresses were assigned geographic coordinates using ArcGIS 9.1 software (ESRI, Redlands, CA) in conjunction with the Dynamap/2000 street network and geocoding database (Tele Atlas, Boston, MA). We calculated proximity to traffic by measuring the distance from geocoded home address to the nearest major roadway (interstate, state, or county highway or major arterial), with a maximum search radius of 150 m. Exposure groups were divided into 0–50 m and > 150 m from the nearest major roadway. These two groups were chosen *a priori* based upon the results of our prior study, which demonstrated the largest associations between traffic exposure and LVM comparing the group residing within 50 m of a major roadway with the group living > 150 m away (Van Hee et al. 2009). This exposure contrast is consistent with the observed distribution of near-roadway air pollutants, with many pollutants rapidly approaching background levels by 150–200 m from large roadways (Hoek et al. 2002; Smargiassi et al. 2005; Zhu et al. 2002).

#### Cardiac MRI imaging

LVM was obtained by cardiac MRI. Images were acquired by 1.5-T MRI scanners (Signa LX and CVi, GE Healthcare, Waukesha, WI; and Somatom Vision and Sonata, Siemens Medical Solutions, Berlin, Germany) using a protocol previously described (Natori et al. 2006). All MRI data were submitted to the MESA MRI Reading Center at Johns Hopkins Hospital for centralized processing using MASS software, version 4.2 (Medis, Leiden, Netherlands).

#### DNA extraction

DNA was extracted from peripheral leukocytes isolated from packed cells of anticoagulated blood by use of a commercially available DNA isolation kit (Puregene; Gentra Systems, Minneapolis, MN). The DNA was quantified by determination of absorbance at 260 nm followed by PicoGreen analysis (Molecular Probes, Inc., Eugene, OR). Two vials of DNA were stored per participant at −70°C and subsequently aliquoted for use.

#### Selection of candidate genes

Twelve genes were selected from among genotyped MESA candidate genes: *ACE*, *ADRB2*, *AGT*, *AGTR1*, *ALOX15*, *EDN1*, *GRK4*, *PTGS1*, *PTGS2*, *TLR4*, *VEGFA*, and *VEGFB*. Genes were chosen based upon hypothesized mechanisms responsible for the effect of air pollutants on LVM as described above.

#### Selection of single-nucleotide polymorphisms

Single-nucleotide polymorphisms (SNPs) were selected in candidate gene loci according to the following criteria: *a*) within the proximal and distal 10-kb regions 5′ and 3′ to the given candidate gene (NCBI Build 35; National Center for Biotechnology Information 2004); *b*) compatibility with the Illumina GoldenGate technology (Fan et al. 2006) as determined by the Assay Design Tool (TechSupport, Illumina, San Diego, CA); and *c*) minor allele frequency (MAF) > 0.05 or a tag (*r*^2^ > 0.8) for another SNP with MAF > 0.05 as determined by applying the multilocus or “aggressive” “Tagger” option of Haploview version 3 (Barrett et al. 2005) using International HapMap project data for CEPH (Caucasian) and Yoruban populations (release 19; International HapMap Consortium 2003). In some cases a complete set of tagged SNPs (tagSNPs) for a given candidate gene was not possible because of these competing criteria. Additional SNPs were added from *a*) LDselect analysis of resequencing information from the SeattleSNPs project if available (Carlson et al. 2004; Nickerson 2003); *b*) nonsynonymous SNPs from dbSNP release 124 (National Center for Biotechnology Information 2005); and *c*) SNPs with prior reports of association proposed by a MESA investigator.

#### Selection of ancestry informative markers

Ancestry informative markers (AIMs) were selected from an Illumina proprietary SNP database to maximize the difference in allele frequencies between any pair of ethnic groups: European American versus African American, European American versus Chinese American, and African American versus Chinese American. Additional markers informative for Mexican-American ancestry were selected from published lists (Choudhry et al. 2006; Collins-Schramm et al. 2004). All subjects were genotyped for the 199 total AIMs selected for inclusion. Principal components of AIMs were calculated using Stata (version 10.1; StataCorp LP, College Station, TX).

#### Genotyping

Genotyping was performed by Illumina Genotyping Services (Illumina Inc.) using their proprietary GoldenGate assay. Of 150 SNPs initially typed, 11 failed quality control measures and were excluded. After excluding 24 SNPs with an overall MAF < 2%, 115 common tagSNPs in the 12 genes under study were chosen for the present analysis. We inferred haplotypes using the expectation-maximization algorithm of PHASE 2.1 (Stephens and Scheet 2005). Haplotype uncertainty was estimated using the diplotype probability. Within a gene, haplotypes with probability-weighted frequencies < 2.5% were grouped together in a gene-specific haplotype bin assigned “others.” A total of 85 common haplotypes were investigated. Supplemental Material, Table 1 (doi:10.1289/ehp.0901535.), summarizes the tagSNPs and haplotypes examined.

#### Data quality control

Illumina performed initial quality control to identify samples and SNPs that failed genotyping according to proprietary protocols and sporadic failed genotypes with GenCall (Illumina) quality score < 99.99%. After removal of failed SNPs and samples, the genotype calling rate was 99.93%, with maximum missing data rate per sample of 2.1% and maximum missing per SNP of 4.98%. The cohort genetic data was checked for cryptic sample duplicates and discrepancies in genetically predicted sex (using X markers) versus study database reported sex. Samples with unresolved duplicate and sex discrepancies were removed from the genetic study database.

### Statistical methods

We produced descriptive statistics using frequencies and percentages for categorical variables and means and SDs for continuous variables. The distributions of covariates were compared across proximity to major roadway categories and log-transformed LVM using analysis of variance methods for continuous variables and chi-square tests for categorical variables. SNP genotype frequencies were tested for race-stratified Hardy–Weinberg equilibrium using exact tests. Race-stratified linkage disequilibrium was assessed for each of the 12 genes under analysis.

A total of 1,139 participants had no missing data on any outcomes or covariates. An initial analysis was performed without imputation and using models with reduced sets of covariates that required no imputation. Subsequently, iterative multiple imputation procedures were performed using switching regression and chained equations for the full 1,376 participants, including data on cotinine levels for 1,082 of 1,376 participants to impute secondhand smoking data (Royston 2007). This process resulted in the full sample of 1,376 adults with some imputed values. The regression analysis was repeated on the imputed sample, yielding overall results similar to those estimated initially. All reported findings are from the imputed data set because the precision of the estimates is improved by the increased sample size, and the full data set is less likely to be subject to bias (Greenland and Finkle 1995; van der Heijden et al. 2006).

We first performed SNP-specific analyses to examine interactions between SNPs and very close (< 50 m) residential proximity to major roadway for log-transformed LVM. We then performed haplotype analyses to also examine interactions between haplotypes and very close proximity to major roadways for LVM. We fitted linear, additive (1 degree of freedom) models separately for each individual SNP (coded 0, 1, and 2 for minor allele copies) and for each haplotype (coded 0, 1, and 2 for number of haplotype copies). Diplotype probabilities were used as weights in the haplotype analyses clustered on participant identifier. Individual *F*-tests for the product term of SNP × proximity < 50 m and haplotype × proximity < 50 m were calculated to obtain *p*-values for interactions. The false discovery rate (FDR) correction (*q*-value) was used to account for multiple testing (115 tests in each SNP model and 85 haplotypes in each haplotype model) (Hochberg and Benjamini 1990; Storey 2002). A *q*-value of 0.2, corresponding to a 20% FDR, was selected as a threshold for acceptable level of significance (Smith et al. 2007). To account for potential confounding by population stratification, we adjusted for the first five principal components of AIMs in all analyses. All models were additionally adjusted for age, sex, height, and weight.

Additional potential confounders for the full model, chosen *a priori* based upon a prior study of the relationship between traffic exposure and LVM (Van Hee et al. 2009), and results of the initial descriptive analyses included household income, highest educational attainment level, systolic and diastolic blood pressure, medication use, low- (LDL) and high- (HDL) density-lipoprotein cholesterol, physical activity, alcohol use, smoking and pack-year history of smoking, secondhand smoke exposure, and diabetes status by fasting blood glucose criteria or medication for diabetes. Medication use was modeled using indicator variables for ACE inhibitors, angiotensin-receptor blockers (ARBs), diuretics, beta-blockers, lipid-lowering medications, aspirin, nonsteroidal antiinflammatory drugs (NSAIDs), and cyclooxygenase (COX) inhibitors. Because several of these covariates (particularly blood pressure) may lie in the causal biological pathway between the exposure and outcome, and as a sensitivity analysis, we also examined several partially adjusted models with reduced sets of covariates. Confidence intervals (CIs) were calculated at an alpha value of 0.05. We then conducted several sensitivity analyses, including stratification by race, blood pressure, and medication use. Subpopulation effects in all interaction models were calculated using linear combinations of predictors in each model (lincom command in Stata).

Data were analyzed using Stata and R (version 2.9; R Development Core Team 2009). The authors had full access to the data and take responsibility for the integrity of the data.

## Results

### Descriptive statistics

Table 1 indicates the overall distribution of participant characteristics. The 1,376 participants in this study ranged in age from 44 to 84 years. Although all MESA participants were free of clinical CVD at baseline, a substantial percentage had cardiac risk factors (Table 1). On bivariate analyses, race, sex, site, antihypertensive medication use, body mass index (BMI), secondhand smoke exposure, and income were at least moderately associated (*p* < 0.2) with proximity to major roadway (data not shown). African Americans, Hispanics, women, participants in New York and Baltimore, those on antihypertensive medication and specifically ACE inhibitor therapy, and those with higher BMIs, higher secondhand smoke exposures, and lower income tended to reside nearer to major roadways. Associations between LVM and nearly every covariate were strong. African-American race, St. Paul study site, male sex, younger age, hypertension, antihypertensive medication use, BMI, blood pressure, cigarette smoke exposure, fasting blood glucose, income, education, lower HDL cholesterol, and higher height and weight were all associated with higher LVM on bivariate analysis. After adjustment for height and weight, LVM was positively associated with age (data not shown).

We noted no significant deviations from race-stratified Hardy–Weinberg equilibrium, such as those associated with genotyping error, on exact testing at an alpha level of 0.05 with Bonferroni correction for 115 tests. Supplemental Material, Table 1 (doi:10.1289/ehp.0901535), lists the selected SNPs and inferred haplotypes for this study, with the frequencies (for tagSNPs) or probability-weighted frequencies (for inferred haplotypes) of each. The principal components of AIMs showed generally good discrimination between self-categorized races, with Hispanics demonstrating the most diverse ancestry and showing some ancestral overlap with other racial groups [Supplemental Material, Figure 2 (doi:10.1289/ehp.0901535)].

### Primary SNP interaction analyses

All SNP–traffic proximity interaction models tested demonstrated significant evidence of interactions for two of the 12 genes tested, *ALOX15* and *AGTR1* [Table 2; see also Supplemental Material, Figure 1 (doi:10.1289/ehp.0901535)]. The minimally adjusted model (model 1) containing only study site and principal components of AIMs, age, sex, height, and weight showed significant (*q* < 0.2) evidence of interactions for rs389566 and rs6801836 in *AGTR1*, with a 9% (95% CI, 2–16%) difference in the impact of traffic proximity (< 50 m residential proximity compared with > 150 m residential proximity) on LVM comparing homozygotes for rs389566 and an 11% (95% CI, 3–19%) difference comparing homozygotes for rs6801836. The full model (model 5) comprising all covariates showed significant evidence of interactions for rs2664593 in *ALOX15* [10% (95% CI, 2–19%) difference in the association between traffic proximity and LVM comparing homozygotes] and rs6801836 in *AGTR1* [9% (95% CI, 2–17%) difference comparing homozygotes]. Complete results of all SNP–roadway proximity interaction tests for all genes are included in Supplemental Material, Table 2 (doi:10.1289/ehp.0901535).

**Figure 1:**
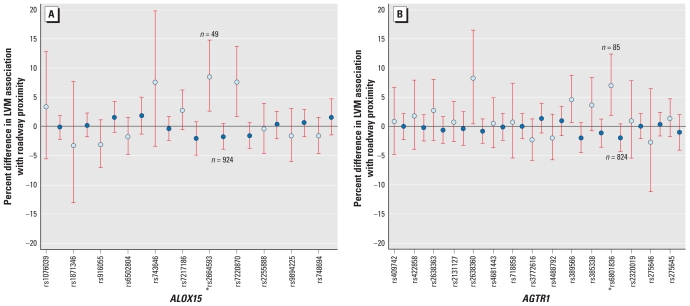
Percent difference in LVM associated with living within 50 m of a major roadway compared with living > 150 m away (fully adjusted model), by all tagSNP homozygotes in *ALOX15* (*A*) and *AGTR1* (*B*). SNPs are arranged in their positional order along the chromosome. Data points indicate estimates for individuals homozygous for the minor allele (light blue) and for the major allele (dark blue). Numbers represent the number of participants in the significant genotype categories. Error bars are 95% CIs. *Interactions meeting *q*-value threshold for statistical significance (*q* < 0.2).

In all cases of significant interactions, the minor allele represented the deleterious allele. The MAF of polymorphisms conferring apparent susceptibility to traffic proximity ranged from 23% (rs6801836) to 33% (rs389566) for *AGTR1* and from 19% (rs2664593) to 21% (rs7220870) for *ALOX15*. The top four SNPs representing greatest evidence for interaction overall in models 4 and 5 consist of one group of two adjacent tagSNPs on *ALOX15* (rs2664593 at position 7 and rs7220870 at position 8) and two nearby tagSNPs on *AGTR1* (rs389566 at position 10 and rs6801836 at position 12). Figure 1 represents the relative location of each evaluated tagSNP on the two genes with significant findings. In the Supplemental Material, Figures 3 and 4 (doi:10.1289/ehp.0901535) show linkage disequilibrium maps for each racial group for *ALOX15* and *AGTR1*. SNP positions 7 and 8 in *ALOX15* and 10–12 in *AGTR1* show strong linkage disequilibrium across all racial groups.

### Haplotype interaction results

After rare haplotypes were collapsed into a single category, remaining haplotypes containing the deleterious SNPs in *AGTR1* and *ALOX15* had low probability-weighted haplotype frequencies [3% for *AGTR1*-E and *AGTR1*-F, 9% for *ALOX15*-C, and 3% for *ALOX15*-I; see Supplemental Material, Table 1 (doi:10.1289/ehp.0901535)]. Despite this reduced power to detect interactions for haplotypes compared with individual SNPs, we found significant (*q* < 0.2) interactions for *AGTR1*-F after taking into account multiple testing. In model 5, proximity to roadway had no significant association with LVM in individuals without this haplotype, whereas the presence of a single haplotype conferred an 8.6% (95% CI, 3.0–14.6%) positive difference in LVM (*p*-value for interaction = 0.002) associated with close (< 50 m) proximity to a major roadway compared with living farther away (> 150 m). All five models showed consistent impacts on susceptibility, with the presence of *AGTR1*-F showing increased susceptibility to traffic proximity on LVM. Complete results of all haplotype–roadway proximity interaction tests for all genes and all models are included in Table 3 of the Supplemental Material (doi:10.1289/ehp.0901535).

### Sensitivity analyses

**Figure 2:**
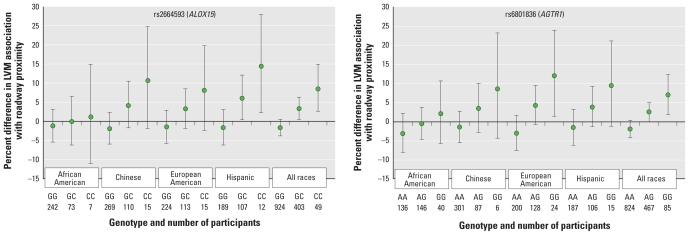
Race-stratified percent difference in LVM associated with living within 50 m of a major roadway compared with living > 150 m away (fully adjusted model) for tagSNPs showing significant evidence of interactive effects: rs2664593 in *ALOX15* (*A*) and rs6801836 in *AGTR1* (*B*). Error bars are 95% CIs.

As described above and shown in Table 2 and Supplemental Material, Figure 1 (doi:10.1289/ehp.0901535), results were not particularly sensitive to model selection. To investigate the impact of racial structure on the findings, we performed stratification by race (using a three-way interaction among SNP, proximity to roadway, and race) for the two significant (*q* < 0.2) interactive SNPs in the full model, an analysis that includes adjustment for the principal components of AIMs (Figure 2). Although we found no significant evidence of a three-way interaction between rs6801836 (*p*-value for three-way interaction = 0.7) or rs2664593 (*p*-value for three-way interaction = 0.6), roadway proximity, and race, the overall results suggest less pronounced gene–environment interactions for African Americans compared with other racial groups.

**Figure 3:**
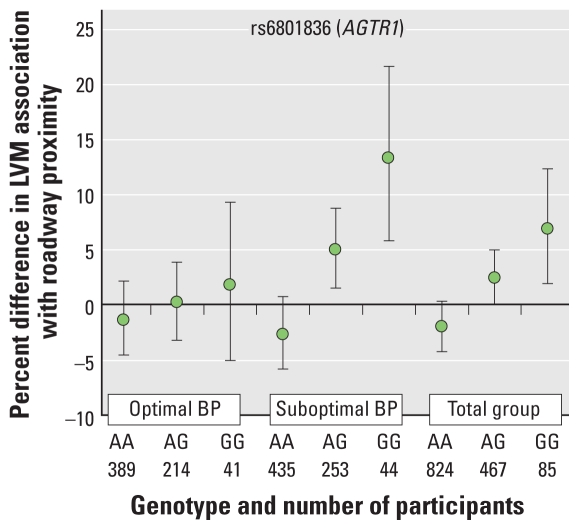
Percent difference in LVM associated with living within 50 m of a major roadway compared with living > 150 m away, by blood pressure (BP) categories (optimal vs. suboptimal) according to JNC VII criteria and rs6801836 (*AGTR1*) genotype. Model comprises SNP, proximity to roadway categories, interaction terms between SNP and proximity, study site, age, sex, height, weight, principal components of AIMs, diabetes, lipids, education, income, alcohol use, physical activity, and smoking. Error bars are 95% CIs.

We also explored stratification by blood pressure categories [Seventh Report of the Joint National Committee on Prevention, Detection, Evaluation, and Treatment of High Blood Pressure (JNC VII) categories for all participants, binary optimal/suboptimal blood pressure, binary hypertensive/not hypertensive] (Chobanian et al. 2003) and use of medications important in the renin-angiotensin system or inflammatory pathways (ACE inhibitors, ARBs, aspirin, and NSAIDs) to determine whether these were groups particularly susceptible to this interaction. Among these analyses, we found significant evidence of interaction among rs6801836, proximity to traffic, and optimal versus suboptimal blood pressure categories. The difference in the LVM change associated with roadway proximity for individuals with greater numbers of the deleterious (G) allele in rs6801836 was significantly larger among individuals with suboptimal blood pressure by JNC VII criteria (systolic blood pressure ≥ 120 mmHg or diastolic blood pressure ≥ 80 mmHg, *n* = 732 participants) than among those with optimal blood pressure (*p*-value for three-way interaction = 0.05; Figure 3). We found no other significant three-way interactions for the medication use or blood pressure categories tested.

## Discussion

We found significant evidence of gene–traffic interactions in MESA, with polymorphisms in *AGTR1* and *ALOX15*, genes important in vascular function and inflammation/oxidative stress, associated with substantial alterations in the association between traffic proximity and LVM. Exploratory analyses additionally revealed that the interaction associated with the common polymorphism in the *AGTR1* gene (rs6801836) is larger for individuals with suboptimal blood pressure (> 120 mmHg systolic or > 80 mmHg diastolic) than for those with optimal blood pressure control. Haplotype analyses, although likely underpowered in this setting, provided additional support for SNP findings in *AGTR1*.

AGTR1 is a well-known regulator of blood pressure and common target of specific pharmacologic intervention (ARB) in hypertension. The specific tagSNP locus (rs6801836) found to demonstrate interactive effects here has not yet been observed to show main effects in MESA or other studies (Bahrami et al. 2008). However, polymorphisms in *AGTR1*, and particularly the A1166C polymorphism (rs5186), have been associated with inflammation (Suchankova et al. 2009) and left ventricular hypertrophy (Smilde et al. 2007). Additionally, an association between the A1166C polymorphism and hypertension has been observed in a number of studies, although results have been somewhat inconsistent (Mottl et al. 2008). A possible explanation for the inconsistencies observed in these studies is the presence of unexamined interactive effects with environmental factors, such as those observed in the present analysis. Our observation that blood pressure and the identified polymorphism in *AGTR1* appear to interact to produce susceptibility to the hypertrophic effects of traffic exposure is consistent with the well-known role of *AGTR1* in the regulation of blood pressure and provides support to the hypothesis that the effect of air pollution is mediated by genes involved in blood pressure regulation. This observation also suggests an important role of both genetic and other environmental factors (in this case, factors that contribute to blood pressure) in modulating the effects of air pollution on CVD.

The protein encoded by *ALOX15* is an oxidizing enzyme that can produce reactive lipid hydroperoxides within the vasculature. Experimental and human epidemiologic studies have demonstrated a range of contrasting effects of *ALOX15* on CVD, and its role at present is not fully understood (Mochizuki and Kwon 2008; Wittwer and Hersberger 2007). Variants specifically at the rs2664593 locus in the 5′ promoter region of the gene have previously (but inconsistently) been associated with carotid atherosclerosis and have been shown to interact in the relationship between carotid wall thickness and insulin resistance (Bevan et al. 2009; McCaskie et al. 2008). In MESA, this specific SNP locus has not shown main effects on LVM (Bahrami H, unpublished observations).

Both *ALOX15* and *AGTR1* are likely to play important roles in inflammation, one of the primary putative mechanisms for the impact of air pollution on CVD and also an important mechanism involved in the development of left ventricular hypertrophy. These genes’ apparent interaction with air pollution to produce increases in LVM could suggest an important role of vascular inflammation in the pathogenesis of traffic-related air pollution–induced CVD more generally.

Linkage disequilibrium mapping of *AGTR1* and *ALOX15* in these analyses suggests that regions near to tagSNPs rs6801836 on *AGTR1* and rs2664593 on *ALOX15* are responsible for the interactions seen in this study. Finer mapping of *ALOX15* and *AGTR1* in these regions may help identify the specific polymorphism associated with these results.

Because the MESA cohort has four distinct racial groups, it represents a unique opportunity to examine gene–environment interactions in a multiethnic population. Analyses performed in this heterogeneous group must take into account the important issue of potential confounding by population stratification. To account for this issue of multiple ethnic groups that also vary in structure by site, we adjusted for both study site and the principal components of AIMs. We additionally examined three-way interactions with race in an analysis that included both site and AIMs and observed no evidence of substantial confounding or effect modification by race. Although we found no significant evidence overall of modification of the interactions by race, the finding that African Americans show apparently less pronounced interactions (Figure 2) bears further investigation. It is possible that additional relationships with other underlying genetic factors that predispose African Americans to known higher LVM (gene–gene interactions) may play a role in attenuating this particular interaction.

Few studies to date have examined gene–environment interactions in the cardiovascular health effects of air pollutants (Baccarelli et al. 2008; Chahine et al. 2007; Park et al. 2006; Schwartz et al. 2005). Of those studies, most have focused on individual functional polymorphisms or individual candidate genes, with few exceptions (Peters et al. 2007; Ren et al. 2010). This is the first study to explore multiple polymorphisms in pathways of interest with the goal of furthering understanding of how air pollution affects cardiac structure specifically.

This study has several limitations. We cannot rule out the possibility that a portion of the findings may have arisen by chance. We chose an FDR of 0.2 as a measure of statistical significance in this study, as have other studies (Smith et al. 2007). By definition, the choice of an FDR threshold to account for multiple testing controls for an expected proportion of incorrectly rejected null hypotheses. Future studies examining these relationships in additional cohorts will be necessary to replicate and confirm these findings. As have other recent studies of the health impacts of air pollution, we used a relatively coarse indicator of exposure to traffic-related air pollution, advantages and disadvantages of which have been described in more detail previously (Van Hee et al. 2009). The exposure misclassification produced by such methods may lead to bias in estimating the effects in subpopulations and overall and may adversely affect standard error estimates (Gryparis et al. 2009; Kim et al. 2009; Szpiro et al. 2009). Because of the chosen exposure metric, this study is unable to distinguish between interactions produced by specific components of traffic-related air pollutants or even non-air pollutants related to traffic (e.g., noise; for more detail, see Van Hee et al. 2009). Ongoing work will help disentangle these traffic-related influences on health.

Although the interactions observed in this study are relatively modest, in this same data set a 10-mmHg increase in systolic blood pressure (one of the factors known to be most important in the development of increased LVM) is associated with only an adjusted 3% higher LVM. Given that individuals homozygous for the deleterious alleles show a 7–8% difference in LVM associated with close proximity to a major roadway, and given the high minor allele frequencies and high prevalence of close residential proximity to a major roadway seen here, the public health impacts of these findings are potentially significant.

In our prior analysis of the relationship between proximity to roadways and VM (Van Hee et al. 2009), we reported results using an untransformed LVM index (rather than log-transformed LVM adjusted for height and weight) as the dependent variable in order to make the results more accessible to the primary audience. As we described in that report, the preferred statistical methods for regression analysis avoid the use of ratios such as the LVM index to prevent spurious correlation (Kronmal 1993). For this reason, and to meet the more stringent requirements of population association studies, which require that the outcome be approximately normally distributed rather than only the residuals (Balding 2006), we have used log LVM adjusted for height and weight in this report. For comparison purposes, we have reported the results of our prior study with the same parameterization used here in Table 4 of the Supplemental Material (doi:10.1289/ehp.0901535).

In addition to replication of these findings specifically, human studies such as pharmacologic intervention trials may further understanding of these potentially modifiable genetic pathways. Although this study was not powered to detect three-way interactions among genes, air pollution, and medication use, the observation that the presence of optimal blood pressure may attenuate the effect of genes and air pollution on cardiovascular outcomes is a provocative one.

As described above, the mechanisms responsible for the effects of air pollutants on CVD remain uncertain, although several studies have implicated inflammation and oxidative stress, as well as impaired vascular function. Modification of the effect of traffic exposures on LVM by genes involved in inflammatory response and vascular function suggests that individuals with genetically determined impaired handling of inflammation and altered vascular responses have greater susceptibility to these effects. This finding lends further support to pathways involving these mechanisms.

## Conclusions

SNPs in genes responsible for vascular function, inflammation, and oxidative stress (*AGTR1* and *ALOX15*) modify associations between proximity to major roadways and LVM. Further understanding of how genes modify effects of air pollution on CVD may help guide research efforts into specific mechanistic pathways.

## Figures and Tables

**Table 1:** Population characteristics at baseline examination: MESA, 2002 (*n* = 1,376).

Characteristic	*n* (%)
Distance to major roadway (m)	
> 150	919 (66.8)
< 50	457 (33.2)

Site/center	
Baltimore, MD	179 (13.0)
Chicago, IL	229 (16.6)
Los Angeles, CA	444 (32.3)
New York, NY	205 (14.9)
St. Paul, MN	150 (10.9)
Winston-Salem, NC	169 (12.3)

Sex	
Female	722 (52.5)

Race/ethnicity	
African American	322 (23.4)
Chinese	394 (28.6)
European American	352 (25.6)
Hispanic	308 (22.4)

Age (years)	
44–54	454 (33.0)
55–64	397 (28.9)
65–74	386 (28.1)
75–84	139 (10.1)

Highest education level completed	
Less than high school	266 (19.3)
Completed high school or some college	437 (31.8)
Technical school or associate degree	178 (12.9)
Bachelor’s degree	255 (18.5)
Graduate/professional degree	237 (17.2)

Gross family income	
< $25,000	463 (33.6)
$25,000 to $49,999	347 (25.2)
$50,000 to $74,999	223 (16.2)
$75,000 to $99,999	120 (8.7)
> $100,000	183 (13.3)

BMI (kg/m^2^)	
< 23	256 (18.6)
23–27.5	540 (39.2)
27.6–40	559 (40.6)
> 40	21 (1.5)

Fasting blood glucose (mg/dL)	
< 100	813 (59.1)
100–125	381 (27.7)
> 125, untreated	56 (4.1)
> 125, treated	124 (9.0)

Cigarette smoking status	
Never	791 (57.5)
Former	415 (30.2)
Current	170 (12.4)

Pack-years smoking	
< 10	1,016 (73.8)
10–19.9	123 (8.9)
≥ 20	230 (16.7)

Secondhand smoke exposure (hr/week)	
0	768 (55.8)
1	158 (11.5)
2–5	130 (9.5)
6–10	50 (3.6)
> 10	92 (6.7)

Alcohol use	
Never	367 (26.7)
Former	287 (20.9)
Current	709 (51.5)

Blood pressure (mmHg)	
< 130/85	863 (62.7)
130–139/85–89	193 (14)
140–159/90–99	241 (17.5)
> 159/99	79 (5.7)
Hypertension medication	473 (34.4)
Angiotensin type 2 antagonists	45 (3.3)
Combinations of angiotensin II antagonists plus diuretics	26 (1.9)
ACE inhibitors without diuretics	146 (10.6)
ACE inhibitors with diuretics	11 (0.8)
Any lipid-lowering medication	209 (15.2)
Aspirin	295 (21.4)
Beta-blockers without diuretics	123 (8.9)
Beta-blockers with diuretics	8 (0.6)
COX-2 inhibitors	89 (6.5)
NSAIDs	183 (13.3)

LDL (mg/dL)	
< 100	390 (28.3)
100–129	535 (38.9)
130–159	320 (23.3)
160–189	94 (6.8)
> 189	17 (1.2)

HDL (mg/dL)	
< 40	291 (21.1)
40–59	768 (55.8)
> 59	315 (22.9)

LVM^a^	
Grams	140.2 ± 37.8
Log LVM	4.9 ± 0.3

Multiple imputation using all data including measured cotinine levels for 1,082 participants was used to impute missing covariates (179 secondhand smoke values, 40 income level measures, 20 LDL and 2 HDL values, 13 alcohol use and 7 pack-years smoking values, and 3 educational level, 3 exercise level, and 2 diabetes status measures).

a Mean ± SD.

**Table 2:** SNP–traffic interactions on LVM, top four interactions by model: percent change in LVM associated with close (< 50 m) residential proximity to major roadway compared with > 150 m, by tagSNP homozygote.

Model/Gene	tagSNP	*p*-Value for interaction	Genotype	Percent difference in LVM associated with traffic proximity (95% CI)
Model 1: study site, age, sex, AIMs, height, weight				
*AGTR1*	rs389566	0.001*	TT	6.3 (2.1 to 10.8)
			AA	−2.9 (−5.5 to −0.2)

*AGTR1*	rs6801836	0.001*	GG	8.6 (3.2 to 14.3)
			AA	−2.5 (−5 to −0.1)

*ALOX15*	rs2664593	0.011	CC	7.5 (1.3 to 14.2)
			GG	−1.6 (−3.9 to 0.7)

*AGTR1*	rs385338	0.012	CC	5.5 (0.8 to 10.5)
			GG	−1.9 (−4.4 to 0.6)

Model 2: model 1 + diabetes, lipids, education, income, alcohol use, and physical activity				
*AGTR1*	rs389566	0.001*	TT	6.1 (1.8 to 10.5)
			AA	−2.9 (−5.5 to −0.2)

*AGTR1*	rs6801836	0.001*	GG	8.5 (3 to 14.2)
			AA	−2.6 (−5 to −0.1)

*ALOX15*	rs2664593	0.013	CC	7.3 (1 to 13.9)
			GG	−1.7 (−4 to 0.7)

*AGTR1*	rs385338	0.018	CC	5.1 (0.4 to 10.1)
			GG	−1.9 (−4.4 to 0.7)

Model 3: model 2 + smoking status, pack-years, and secondhand smoke exposure				
*AGTR1*	rs389566	0.002*	TT	6 (1.7 to 10.4)
			AA	−2.8 (−5.4 to −0.2)

*AGTR1*	rs6801836	0.001*	GG	8.2 (2.8 to 14)
			AA	−2.5 (−5 to −0.1)

*ALOX15*	rs2664593	0.013	CC	7.3 (1 to 13.9)
			GG	−1.7 (−4 to 0.7)

*AGTR1*	rs385338	0.019	CC	5.1 (0.3 to 10)
			GG	−1.9 (−4.4 to 0.7)

Model 4: model 3 + blood pressure				
*AGTR1*	rs6801836	0.002*	GG	7.7 (2.6 to 13.1)
			AA	−2 (−4.3 to 0.4)

*ALOX15*	rs2664593	0.004*	CC	8.3 (2.4 to 14.7)
			GG	−1.5 (−3.7 to 0.8)

*AGTR1*	rs389566	0.007	TT	5.1 (1.1 to 9.3)
			AA	−2 (−4.5 to 0.6)

*ALOX15*	rs7220870	0.013	AA	7.3 (1.4 to 13.5)
			CC	−1.3 (−3.5 to 1)

Model 5 (*a priori* model): model 4 + medication use				
*ALOX15*	rs2664593	0.003*	CC	8.5 (2.6 to 14.9)
			GG	−1.7 (−3.9 to 0.5)

*AGTR1*	rs6801836	0.004*	GG	7 (1.9 to 12.4)
			AA	−1.9 (−4.2 to 0.4)

*ALOX15*	rs7220870	0.008	AA	7.5 (1.7 to 13.8)
			CC	−1.5 (−3.7 to 0.7)

*AGTR1*	rs389566	0.013	TT	4.6 (0.6 to 8.8)
			AA	−1.9 (−4.4 to 0.6)

Model 1 (minimally adjusted model) contains each tagSNP, proximity to roadway categories, interaction terms between SNP and proximity, study site, age, sex, height, weight, and the principal components of AIMs. Model 2 contains covariates from model 1, plus additional CVD risk factors (diabetes, lipids, education, income, alcohol use, and physical activity) except smoking. Model 3 contains covariates from model 2 plus smoking. Model 4 adds blood pressure to model 3. Model 5, the fully adjusted model, adds medication use (ACE inhibitors, ARBs, diuretics, beta-blockers, lipid-lowering medications, aspirin, NSAIDs, and COX inhibitors) to model 4.

* Interactions meeting *q*-value threshold for statistical significance (*q* < 0.2).
